# High IL-17E and Low IL-17C Dermal Expression Identifies a Fibrosis-Specific Motif Common to Morphea and Systemic Sclerosis

**DOI:** 10.1371/journal.pone.0105008

**Published:** 2014-08-19

**Authors:** Paola Adele Lonati, Nicolò Costantino Brembilla, Elisa Montanari, Lionel Fontao, Armando Gabrielli, Serena Vettori, Gabriele Valentini, Emmanuel Laffitte, Gurkan Kaya, Pier-Luigi Meroni, Carlo Chizzolini

**Affiliations:** 1 Immunology and Allergy, University Hospital and School of Medicine, Geneva, Switzerland; 2 Experimental Laboratory of Immunological and Rheumatologic Researches, IRCSS Istituto Auxologico Italiano, Milan, Italy; 3 Dermatology, University Hospital and School of Medicine, Geneva, Switzerland; 4 Institute of Clinica Medica, Ancona, Italy; 5 Rheumatology, Department of Clinical and Experimental Medicine, Second University of Naples, Naples, Italy; 6 Division of Rheumatology, Department of Clinical Sciences and Community Health, University of Milan, Milan, Italy; University of Texas Health Science Center at Houston, United States of America

## Abstract

**Background:**

High interleukin (IL)-17A levels are characteristically found in the skin of systemic sclerosis (SSc) individuals. Our aim was to investigate whether the dermal expression of IL-17A and related IL-17 family members (i.e. IL-17C, IL-17E and IL-17F) could distinguish fibrotic from healthy skin and could show similarities in SSc and morphea, two disorders with presumed distinct pathogenesis, but characterized by skin fibrosis.

**Methods:**

Biopsies were obtained from the involved skin of 14 SSc, 5 morphea and 8 healthy donors (HD) undergoing plastic surgery. Immunohistochemistry/immunofluorescence techniques were coupled to a semi-automated imaging quantification approach to determine the presence of the IL-17 family members in the skin. The in vitro effects induced by the IL-17 family members on fibroblasts from normal and SSc individuals were assessed by ELISA and RIA.

**Results:**

Positive cells for each of the IL-17 isoforms investigated were present in the dermis of all the individuals tested, though with variable frequencies. SSc individuals had increased frequency of IL-17A+ (p = 0.0237) and decreased frequency of IL-17F+ (p = 0.0127) and IL-17C+ cells (p = 0.0008) when compared to HD. Similarly, morphea individuals had less frequent IL-17C+ cells (p = 0.0186) in their skin but showed similar number of IL-17A+ and IL-17F+ cells when compared to HD. Finally, IL-17E+ cells were more numerous in morphea (p = 0.0109) and tended to be more frequent in SSc than in HD. Fibroblast production of IL-6, MMP-1 and MCP-1 was enhanced in a dose-dependent manner in the presence of IL-17E and IL-17F, but not in the presence of IL-17C. None of the cytokine tested had significant effect on type I collagen production. Of interest, in SSc the frequency of both IL-17A and IL-17F positive cells increased with disease duration.

**Conclusions:**

The frequency of IL-17A and IL-17F distinguish SSc to morphea individuals while dermal expression of IL-17C (low) and IL-17E (high) identifies a fibrosis-specific motif. The specific IL-17C/IL-17E cytokine combination may thus play a role in the development of fibrosis.

## Introduction

Skin fibrosis is a non-physiological process characterized by excessive deposition of extracellular matrix (ECM) accompanied by impaired ECM degradation and turnover. It is caused by the transition of quiescent fibroblasts into activated myofibroblasts, which characteristically overproduce different collagen types, other ECM components, and have defective production of collagen-digesting enzymes [1], [2]. Skin fibrosis is the leading manifestation of systemic sclerosis (SSc) and localized forms of scleroderma including morphea. SSc is a systemic inflammatory disorder characterized by widespread vascular abnormalities, with limited cutaneous (lcSSC) and diffuse cutaneous (dcSSc) involvement usually segregating with specific autoantibodies. The gastrointestinal tract, lungs, heart and kidneys are frequently affected [1], [3]. Morphea is a fibrosing condition limited to the skin and subcutaneous tissues, eventually underlying bones and, rarely, the central nervous system [4]. While systemic sclerosis and morphea share physiopathological similarities [5], they may be distinguished both at histological and molecular levels [6], [7], thus highlighting exquisitely specific differences.

Cytokines are thought to play a role in the initiation and/or maintenance/amplification of fibroblast deregulation [2]. Recently, interleukin (IL)-17 has attracted interest and found to be mechanistically involved in animal models of fibrosis. Thus, IL-17A was shown to participate to bleomycin-induced lung and skin fibrosis, IL-17A deficiency attenuated skin thickness in tight skin-1 mice and neutralization of IL-17A inhibited silica-induced chronic inflammation and pulmonary fibrosis [8]–[11]. While increased number of Th17 cells or elevated levels of IL-17A have been reported by many investigators in the peripheral blood [12], [13], bronchoalveolar lavage fluid [14] and the dermis of SSc individuals [15], the available data in humans do not unanimously point to a clear pro-fibrotic role of IL-17A (reviewed in [16]). In the one hand, IL-17A has been shown to enhance dermal fibroblast proliferation, ICAM expression, IL-6, IL-8, monocyte chemoattractant protein (MCP)-1 and matrix metalloproteinase (MMP)-1 production [17]–[22]. In the other hand, IL-17A has been reported to inhibit collagen production and alpha-smooth muscle actin expression induced by transforming growth factor (TGF)-β [15], [23]. Moreover, an inverse correlation between skin thickness and IL-17A+ cell numbers in SSc skin is evidence supporting an anti-fibrotic activity of IL-17A [15].

IL-17A is the founding member of the eponym cytokine family, which includes: IL-17B, IL-17C, IL-17D, IL-17E (also known as IL-25) and IL-17F. IL-17F shares 44% amino acids with IL-17A, whereas the other members share a more limited 15–27% identity. IL-17A and IL-17F can be secreted as disulfide-linked homo- or hetero-dimers and bind the same IL-17RA IL-17RC heterodimeric receptor [24], [25]. In general, IL-17F shares functions with IL-17A, but at variance with IL-17A, IL-17F did not inhibit collagen production induced in fibroblasts by TGF-β [23], although both IL-17A and IL-17F modulated the function of fibrocytes upon CD40 engagement [26]. IL-17C binds the IL-17RA/IL-17RE heterodimer, acts primarily on epithelial cells enhancing the production of antibacterial peptides and on T cells enhancing T helper (Th)17 response [27]. IL-17E binds to the IL-17RA/IL-17RB heterodimer and plays important roles in favoring and participating to Th2 responses [28]. While fibroblasts have been reported not to respond to IL-17C, IL-17E was shown to enhance collagen production by lung fibroblasts [29] and to be overexpressed in individual presenting with idiopathic pulmonary fibrosis [30]. IL17E-dependend production of IL-13 by type 2 innate lymphoid cells (ILC2) is critical in the generation of pulmonary fibrosis in experimental mouse models [30].

Our aim was to investigate whether the proportion of cells producing IL-17F, IL-17C and IL-17E in addition to IL-17A could segregate differentially in normal and fibrotic skin. Our results highlight a fibrotic skin-specific pattern common to SSc and morphea characterized by low IL-17C and high IL-17E. However, IL-17A was found to be high and IL-17F low specifically in SSc compared to morphea, thus pointing to substantial differences between these two disorders. IL-17E and IL-17F, but not IL-17C, promoted in vitro a pro-inflammatory fibroblast phenotype with limited impact on collagen production.

## Materials and Methods

### Individuals

Fourteen SSc and 5 clinically diagnosed morphea individuals were prospectively included. SSc patients met the American Rheumatism Association diagnostic criteria for SSc and classified according to Le Roy et al. [31]. Clinical characteristics of all the individuals are summarized in Table 1. A biopsy was performed in the affected skin of SSc and morphea individuals. The control group (HD) consisted of 8 age and sex matched individuals, 5 underwent corrective breast or abdominal surgery while 3 underwent excisional biopsy in which perilesional healthy skin was used. Skin biopsies were divided in two fragments, one included in paraffin for immunohistochemical processing and the other used for *ex vivo* fibroblast generation. None of the healthy individuals had dermatological disorders and none was under immunosuppressive agents or glucocorticoids at the time of sampling. This study was approved by the ethical committee of the institutions involved (Comité departemental de médicine interne et médicine communautère des Hôpitaux Universitaires de Genève, Geneva, Switzerland; and the Institutional Review Board of the Istituto G. Pini, Milan, Italy) and was conducted according to the Declaration of Helsinki. Written informed consent was obtained from each individual.

**Table 1:** Clinical characteristics of the study populations.

		SSc	morphea
n		14	5
male/female	(n)	2/12	1/4
age, years	(median/range)	59.5/31–76	41/24–68
disease duration, months	(median/range)	71/5–180	5/3–7
Disease subsets	(n, limited/diffuse)	4/10	n/a
MRRS	(median/range)	15/5–29	n/a
ANA	(n)	14	0
ATA	(n)	8	n/a
ACA	(n)	2	n/a
synovitis	(n)	4	0
GERD	(n)	7	0
myositis	(n)	3	0
DU	(n)	4	0
tendon friction rubs	(n)	5	0
ILD	(n)	4	0
Prednisone, mg/d	(median/range)	4.5 (0–17.5)	0
other immunosuppressants	(n)	2	0

ACA: anti-centromere antibody; ANA: anti-nuclear antibody; ATA: anti-topoisomerase-1 antibody; DU: digital ulcer; GERD: gastro-esophageal reflux disease; ILD: interstitial lung disease; MRRS: Modified Rodnan Skin Score; n/a not available.

### Reagents

Polyclonal goat anti-human IL-17A Ab, mouse anti-human IL-17C, IL-17E and IL-17F mAbs, recombinant human tumor necrosis factor (TNF), transforming-growth factor (TGF), IL-17A, IL-17C, IL-17E and IL-17F were from R&D Systems (Abingdon, UK); mouse anti-human mast cell tryptase mAb (clone AA1), polyclonal rabbit anti-mouse and goat anti-mouse immunoglobulins biotinylated from Dako (Glostrup, DK); monoclonal mouse anti-human CD3 (clone PS1) from Novocastra (Newcastle, UK); Alexa-488-conjugated donkey anti-goat and alexa-568 conjugated donkey anti-mouse or anti-rabbit, Dulbecco's modified Eagle's medium (DMEM), phosphate buffered saline (PBS) glutamine, penicillin, streptomycin, trypsin, sodium pyruvate and fetal calf serum were from Life technologies (Paisley, UK). Tyramide, 3,3-Diaminobenzidine (DAB), α-ketoglutaric acid, β-amino propionitrile and L-ascorbic acidwere from Sigma (St. Louis, MO); Vectastain elite ABC kit and Vectashield with DAPI from Vectorlab (Peterborough, UK). All the antibodies have been used at the final concentration of 2.5 µg/ml.

### Fibroblast culture

Fibroblast cell lines were generated after 0.1% type I collagenase digestion at 37°C for 2 hours of skin biopsies as previously reported [15]. Adherent cells were grown in DMEM containing 1% nonessential amino acids, 1% L-glutamine, 1% sodium pyruvate, 50 U/ml penicillin, 50 µg/ml streptomycin and 10% FCS. All experiments were performed with fibroblasts within passage 8. Fibroblasts were seeded at 2×104 cell/well in triplicates in 96-wellplates, serum-starved overnight and incubated with the indicated reagents in DMEM containing 1% FCS, 25 µg/ml L-ascorbic acid, 3.4 µg/ml α-ketoglutaric acid and 50 µg/ml β-amino propionitrile to favor collagen maturation as described [32]. IL-17A was added at 30 ng/ml, IL-17C, IL-17E and IL-17F were added at a concentration ranging from 3 to 600 ng/ml, TGF-β at 10 ng/ml, TNF at 1 ng/ml. Supernatants were harvested at 48 h and frozen until protein determination.

### Chemokine, cytokine and collagen assays

MCP-1, MMP-1 and IL-8 were quantified in culture supernatants by ELISA duoset kit (R&D), IL-6 by eBioscience (San Diego, CA). Collagen production was assessed by RIA quantification of PINP (Orion Diagnostica, Finland) according to the manufacturer instructions.

### Immunohistochemistry (IHC)

After being deparaffinized in xylene and absolute ethanol with the Shandon Varistain Gemini Slide Stainer (Global Medical Instrumentation Inc., Ramsey, MN), tissue sections were placed in a box filled with the appropriate epitope damasking buffer (10 mM citrate buffer/1 mM EDTA pH 7,5 for anti-IL-17A, anti IL-17E, anti-tryptase and anti-CD3 antibodies; 10 mM Tris buffer/1 mM EDTA pH 9 for anti-IL-17C and anti-IL-17F antibodies), and heated in a microwave at 600 W for 20 min. Endogenous peroxidase activity was inhibited by treatment with 0.9% hydrogen peroxide in water for 20 min at room temperature. The tissue sections were incubated for 1 h with primary antibodies diluted in PBS-0.3% BSA, washed twice with PBS and incubated for 30 min with biotinylated secondary antibodies diluted in PBS-0.3% BSA. Immune reactivity was detected with the Vectastain ABC kit using DAB as substrate. Subsequently, slides were counterstained with hematoxylin using the Shandon Varistain Gemini Slide Stainer and mounted with the ClearVue Coverslipper (Thermo Scientific, Waltham, MA). Immunohistochemical stainings were acquired with the Mirax-midi scan microscope (Carl Zeiss Microscopy GmbH, Oberkochen, DE). In negative controls primary antibodies were omitted.

### Indirect immunofluorescence (IIF)

After paraffin removal and epitope retrieval, tissue sections were incubated with primary antibodies. The binding was revealed with Alexa Fluor-488, or Alexa Fluor-568 conjugated anti-mouse or anti-goat secondary antibodies. The slides were mounted with Vectashield with DAPI. Laser-scanning confocal images were acquired using the LSM510meta and LSM700 microscope (Zeiss).

Negative controls, stained only with secondary antibodies, did not result in significant fluorescence and were omitted from the figures.

### Quantification of IHC or IIF images

IHC images were acquired by scanning the whole tissue sections using the Mirax-midi scan microscope. At least two sections, having a mean area of 11.9 mm^2^, were analyzed per individual. Each section was manually subdivided using the Panoramic Viewer Software (3DHISTECH, Budapest, Hungary) in three distinct areas:epidermis, dermis (including the superficial and the deep one) and subcutaneous tissue. Annexes were excluded from the analysis. A semi-automated method to quantify the number of IL-17A positive cells in the dermis of all individuals included in the study was developed using the Metamorph/MetaXpress software (Molecular Devices, PA, USA). Brown elements (positive cells) with size limits were counted and normalized to total number of cells (blue elements) in each field. IIF images were obtained with the LSM700 microscopes at 40x magnifications and processed with the Metamorph/MetaXpress software. A threshold for positivity was defined for each channel. DAPI-staining (blue) was used to determine a size limit for subsequent analysis and to create a mask that was applied to the green and the red channels. The resulting images were semi-automatically processed with Integrated Morphometry Analysis tool, where number and area parameters were measured. At least 6 fields of 0.044 mm^2^ per individual were analyzed. The results were expressed as percentage of positive cells over total cells.

To verify the coherence of the results, representative images were manually reviewed by one of us (PAL).

### Statistical analysis

Statistical analysis was performed using GraphPad Prism version 6.0 (Graphpad Software, La Jolla, California, USA). Significant difference between samples was computed using Mann-Whitney or one-sample t test. Correlation was assessed by Pearson test. P values <0.05 were considered statistically significant.

## Results

### Dermal expression of IL-17A and IL-17F distinguish SSc from morphea

**Figure 1:**
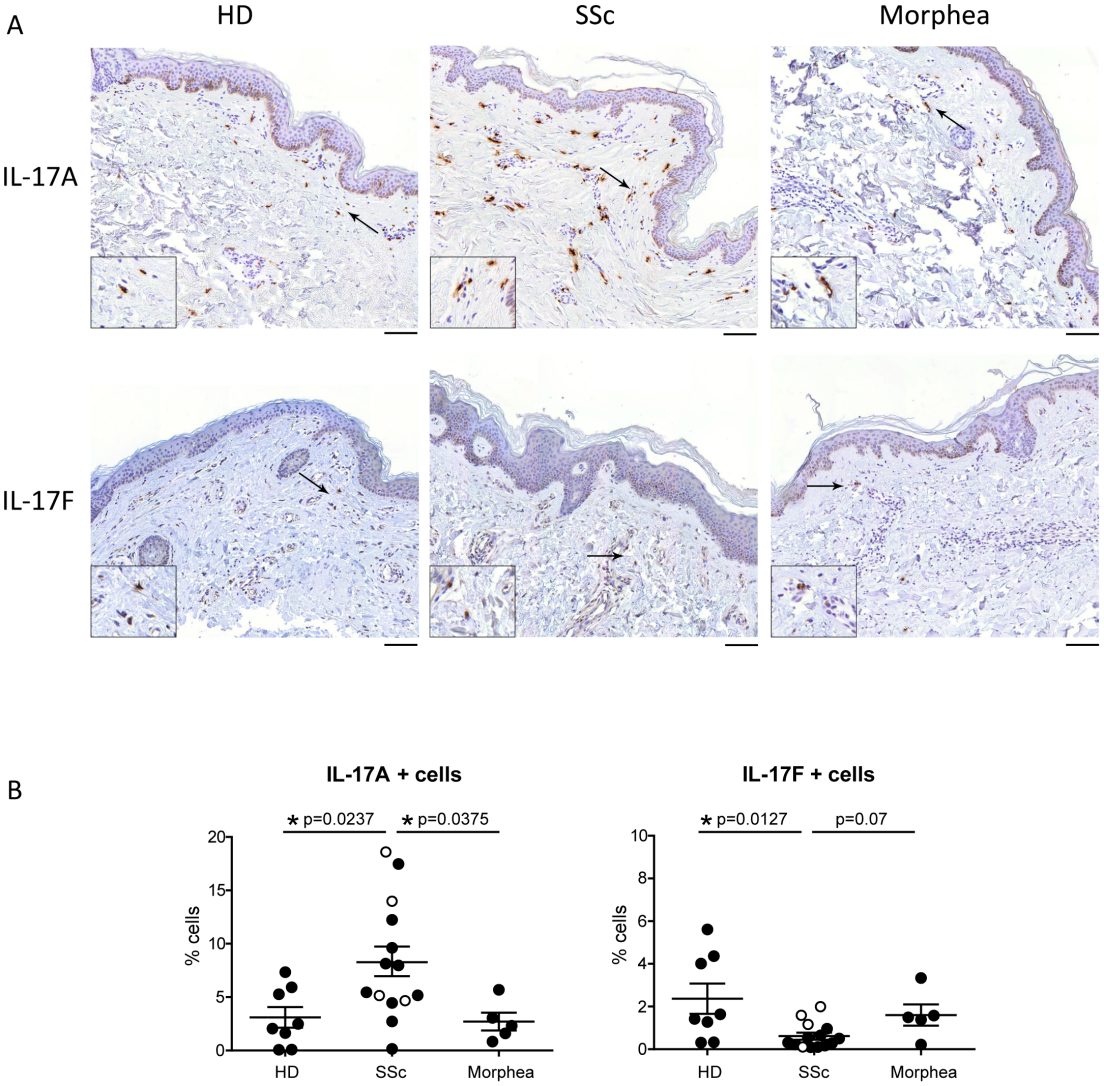
Frequency of IL-17A+ and IL-17F + cells in SSc and morphea. **A.** Immunohistochemical analysis of IL-17A and IL-17F in healthy (HD), lesional SSc and morphea skin. Arrows indicate IL-17A (upper panels) or IL-17F (lower panels) positive cells. Results are representative of 14 SSc, 5 Morphea and 8 HD individuals. Original magnification 20X, scale bar 100 µm. Insets, 2X. **B.** Frequency of IL-17A+ and IL-17F+ cells expressed as percentage of total cells. Each symbol represents a distinct individual and the line depicts the mean. Empty and full dots refer to limited and diffuse SSc, respectively. Significance was assessed by Mann-Whitney test.

**Figure 2:**
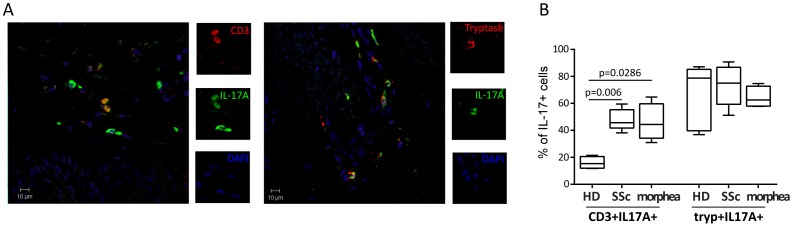
Presence of CD3+IL-17+ T cells and tryptase+IL17+ mast cells in morphea. **A.** Indirect immunofluorescence analysis was used to assess the expression of IL-17A (green), in combination with CD3 (red, left panel) or Tryptase (red, right panel) and DAPI staining of nuclei (blue) in the dermis of a representative of 5 morphea biopsies. Original magnification 40X. **B.** Box-plots show the quantification of CD3+IL-17A+ and Tryptase+IL-17A+ cells expressed as percentage of total IL-17A+ cells in 4 HD, 4 SSc and 4 morphea skin section. The box represents values between 25^th^ and 75^th^ percentile with a line at the median (50^th^ percentile). The whiskers extend above and below the box to show the highest and the lowest values.

IL-17A and IL-17F share high homology and are thought to be coordinately expressed [33], [34]. We first aimed at assessing the frequency of these two isoforms in SSc, morphea, and HD. IL-17A+ cells were significantly more numerous in 14 SSc than 8 HD (p = 0.0237), thus extending our previous results on 8 of these 14 individuals [15] (Figure 1A and B, upper panels). In contrast, and much to our surprise, the frequency of IL-17F + cells was significantly lower in the dermis of systemic sclerosis when compared to healthy skin (p = 0.0127) (Figure 1A and B, lower panels). When comparing dSSc and lSSc, we found no statistically significant differences in the frequency of IL-17A+ and IL-17F+ cells (Figure 1B). However, a trend was observed for IL-17F, with 3 out of 4 lSSc having an IL-17F frequency higher than the 75 percentile of those of dSSc individuals (Figure 1B) The expression pattern of these IL-17 isoforms was specific to SSc, since the frequency of IL-17A+ and IL-17F + cells identified in the skin of 5 morphea patients was similar to that found in HD skin (Figure 1A and B). The specificity of these findings was confirmed by the absence of background staining in the presence of negative control antibodies. IL-17A and IL-17F expressing cells were evenly distributed across the superficial and deep dermis regardless of the disease condition. IL-17A+ cells were modestly more numerous than IL-17F+ cells in HD (1.3 fold) and morphea (1.7 fold), but substantially more in SSc (13.6 fold) (Figure 1B). No IL-17A or IL-17F positive cells were found in the epidermal layer (Figure 1A). CD3+ T cells and tryptase+ mast cells were the main cell types expressing IL-17A in morphea and in SSc [15], with no difference in their relative frequency between the two fibrotic disorders (Figure 2).

### Dermal expression of IL-17C and IL-17E identifies a fibrosis-specific motif

**Figure 3:**
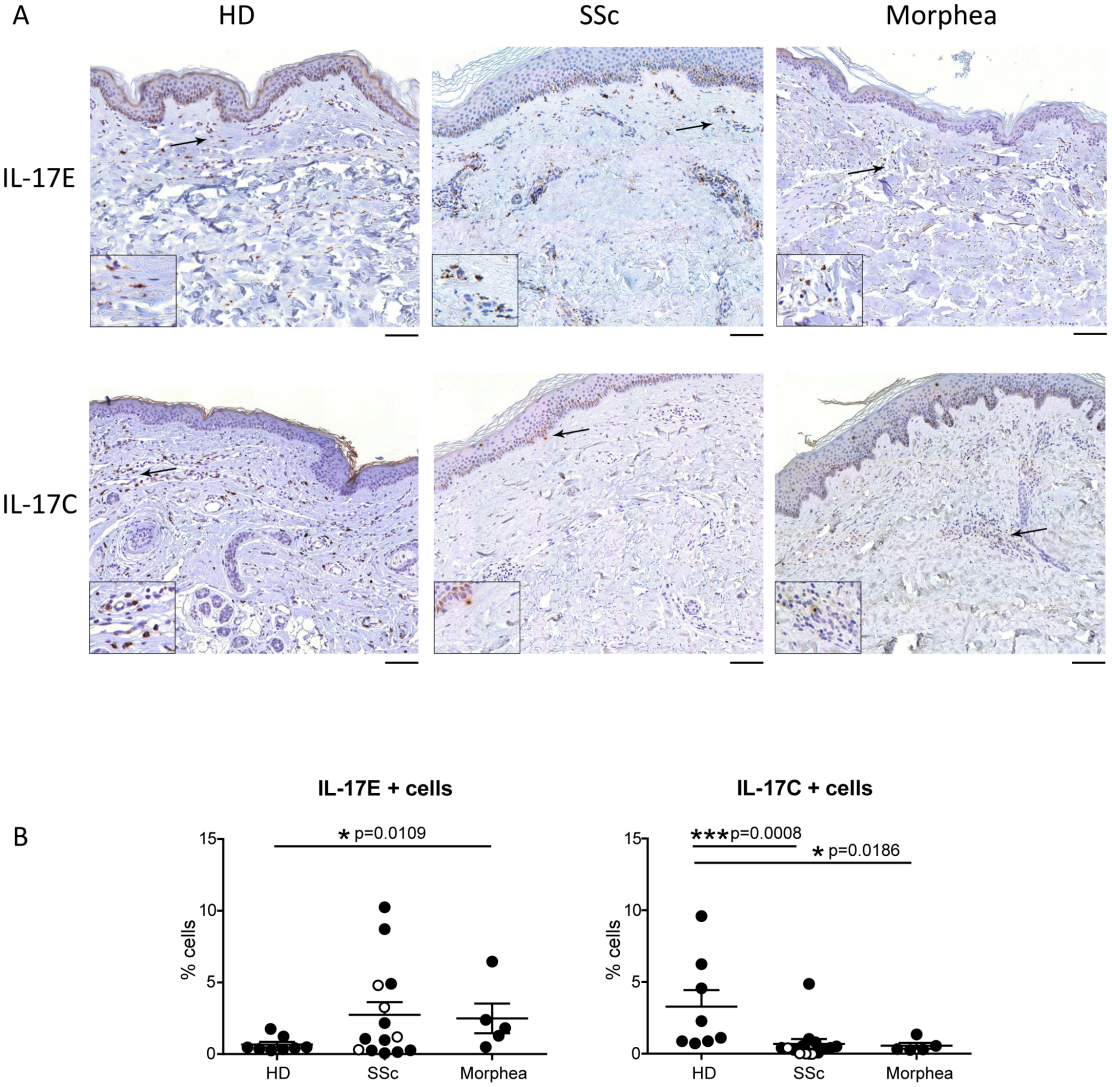
High frequency of IL-17E+ and low frequency of IL-17C + cells identifies a fibrosis-specific motif common to SSc and morphea. **A.** Immunohistochemical analysis for IL-17C and IL-17E in healthy (HD), lesional SSc and morphea skin. Arrows indicate IL-17E (upper panels) or IL-17C (lower panels) positive staining. Results shown are representative of 14 SSc, 5 Morphea and 8 HD individuals. Original magnification 20X, scale bar 100 µm. Insets, 2X. **B.** Frequency of IL-17E+ and IL-17C+ cells expressed as percentage of total cells. Each symbol represents a distinct individual and the line depicts the mean. Empty and full dots refer to limited and diffuse SSc, respectively. Significance was assessed by Mann-Whitney test.

We next assessed whether IL-17C and IL-17E were differentially expressed in HD, SSc and morphea. IL-17C+ cells were detected in all skin samples and were located and evenly distributed mainly in the dermis (Figure 3A, upper panels). However, the frequency of IL-17C+ cells was dramatically lower in the dermis of SSc (p = 0.0008) and morphea (p = 0.0186) when compared to HD (Figure 3B, upper panels). IL-17E+ cells were identified in the dermis of all samples and tented to preferentially aggregate into inflammatory infiltrates, especially in SSc (Figure 3A, lower panels). IL-17E+ cells were significantly more abundant in morphea (p = 0.0109) and had a clear tendency to be more numerous in SSc than in HD (Figure 3B, lower panels). Thus, in 8 of 14 SSc samples the frequency of IL-17E+ cells was above the 75 percentile of those found in HD. Together, our findings show that SSc and morphea share high IL-17E+ and low IL-17C+ cell frequencies, thus defining a cytokine expression pattern which may represent a fibrotic skin-specific motif.

### IL-17 isoforms are not coordinately expressed in fibrotic skin

**Figure 4:**
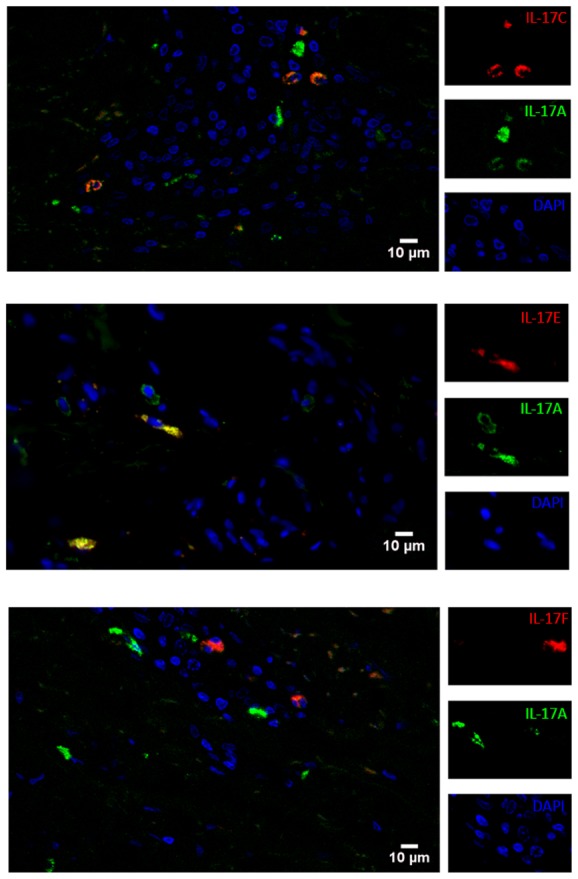
Co-expression of IL-17A with IL-17C or IL-17E. Indirect immunofluorescence analysis was used to assess the expression of IL-17A (green), in combination with IL-17C (**A**) or IL-17E (**B**) or IL-17F (**C**) (red), and DAPI staining for nuclei (blue) in the dermis of one biopsy of 3 assessed (1 HD, 1 SSc and 1 morphea). Original magnification 40X.

We then addressed the question whether the IL-17 isoforms were co-regulated or not in fibrotic skin. To this aim we determined by double staining whether IL-17A+ cells co-expressed another family member at the single cell level. Of interest, we observed some IL-17A/IL-17C and IL-17A/IL-17E double positive cells (Figure 4), but practically we did not observe any IL-17A/IL-17F double positive cells. Thus, within the IL-17A+ cells compartment 12–28% co-expressed IL17C, 20–41% co-expressed IL-17E, but only 0–3% co-expressed IL-17F. Reciprocally, within IL-17C+ cells 33–50% co-expressed IL-17A, within IL-17E+ cells 33–46% co-expressed IL-17A and within IL-17F+ cells only 0–10% co-expressed IL-17A. To further circumstantiate these findings, we checked whether IL-17A+ cells frequencies correlated with the frequencies of cells expressing IL-17C, IL-17E and IL-17F identified by single staining and found no statistical association whether in SSc or HD (data not shown). Similarly, no correlation was found when analyzing all possible combinations of IL-17 isoforms whether in SSc or HD, thus indicating that IL-17 family members are independently expressed in the skin in general and in fibrotic skin in particular.

### The frequency of IL-17A+ and IL-17F+ cells correlates with disease duration

**Figure 5:**
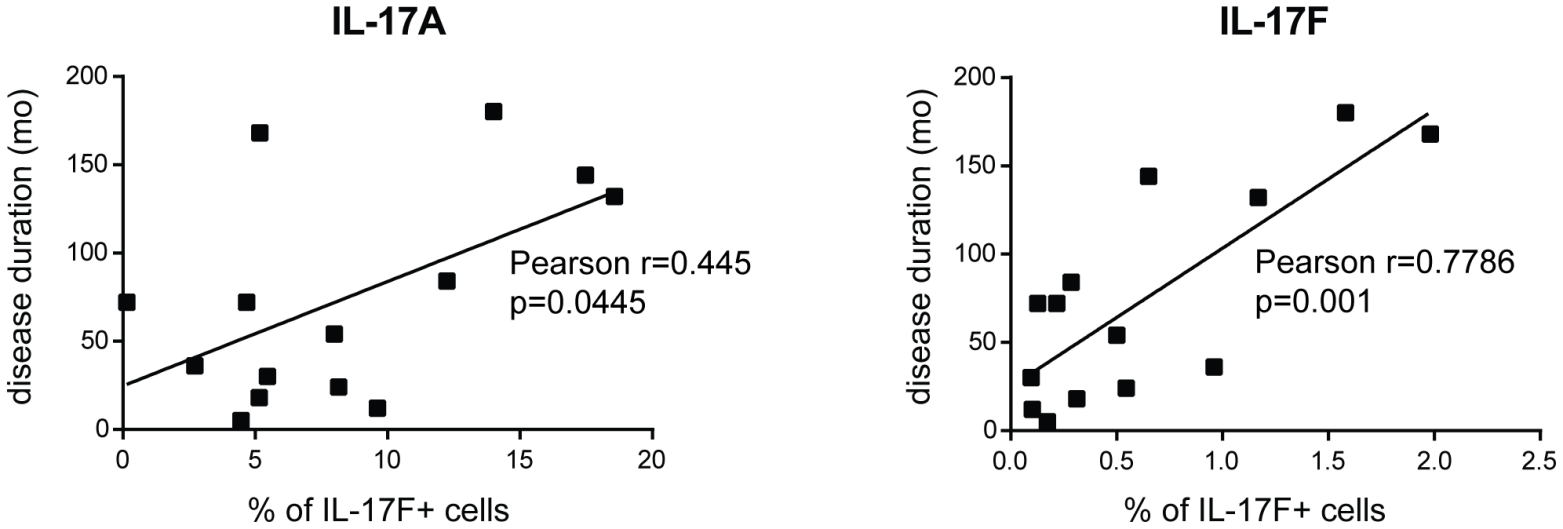
IL-17A+ and IL-17F+ cell frequency positively correlates with disease duration. IL-17A+ and IL-17F+ cells were identified by immunohistochemistry. Each symbol represents a single individual. Significance was assessed by Pearson's correlation test. mo  =  months.

We then sought to examine whether the frequency IL-17 positive cells would correlate with some clinical characteristics. Statistically significant, positive correlation was found between the frequency of IL-17A+ and IL-17F+ cells and disease duration (Figure 5). Furthermore, the frequency of IL-17F+, but not IL-17A+ cells correlated inversely with the modified Rodnan skin score (MRSS) (Figure 5). No correlation with disease duration and skin score was found with the frequency of IL-17C+ and IL-17E+ cells (not shown).

### IL-17F and IL-17E, but not IL-17C, promote a pro-inflammatory fibroblast phenotype in normal and SSc individuals

**Figure 6:**
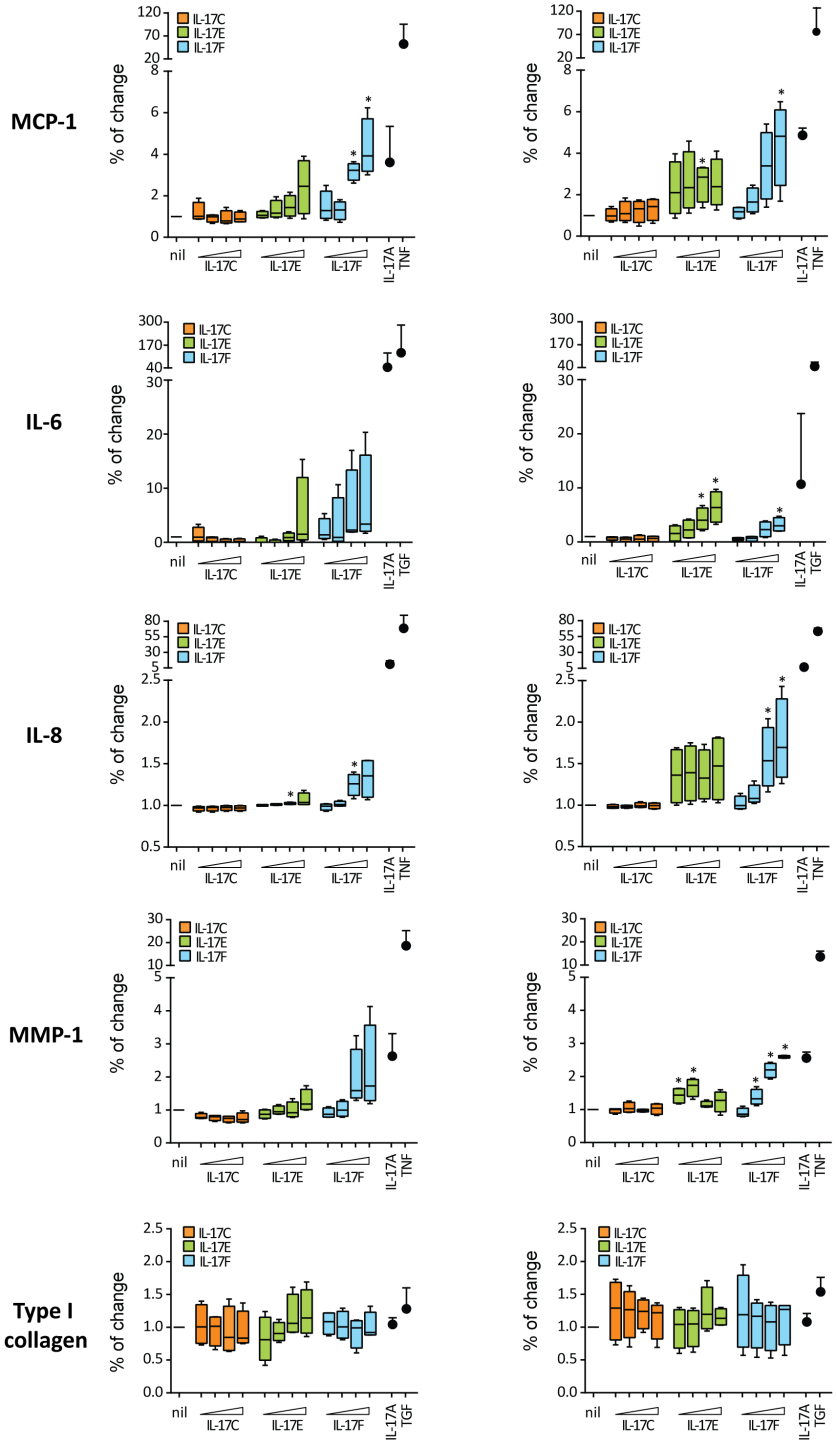
IL-17F and IL-17E, but not IL-17C, enhance MCP-1 and MMP-1 production by normal and SSc fibroblasts. Dermal fibroblasts were cultured in triplicate in the presence of increasing amounts (3, 30, 300, 600 ng/ml) of IL-17C, IL-17E or IL-17F for 48 h. Box-plot show the levels of MCP-1, IL-6, IL-8, MMP-1 and type I collagen assessed in fibroblast culture supernatants from 4 HD and 4 SSc. The box represents values between 25^th^ and 75^th^ percentile with a line at the median (50^th^ percentile). The whiskers extend above and below the box to show the highest and the lowest values. IL-17A (30 ng/ml), TNF (A, 1 ng/ml) or TGF-β (B, 10 ng/ml) were used as controls. Significance versus nil condition was assessed by one-sample t-test.

Several lines of evidence indicate that IL-17A in humans promotes fibroblast pro-inflammatory responses while inhibiting fibrosis [15], [17]–[23]. We therefore investigated whether other IL-17 family members could modulate dermal fibroblasts from SSc and HD individuals to produce inflammatory cytokines and type I collagen. IL-17F dose-dependently enhanced the production of monocyte chemoattractant protein (MCP)-1, IL-6, IL-8 and matrix metalloproteinase (MMP)-1 to a similar extent in fibroblasts from 4 SSc and 4 HD (Figure 5). It should be noted that to induce the production of similar amounts of MCP-1 and MMP-1 ten times more IL-17F than IL-17A was needed (Figure 6). IL-17E dose-dependently increased the production of MCP-1 and MMP-1, especially in HD (Figure 6) and IL-6, especially in SSc fibroblasts. The cytokine levels induced by IL-17E were largely below those induced by similar concentration of IL-17A. IL-17C did not induce MCP-1, IL-6, IL-8 and MMP-1 production by dermal fibroblasts (Figure 6). We also assessed whether IL-17C, IL-17E and IL-17F could modulate the production of type I collagen, but we could not detect any significant change in HD and SSc fibroblasts (Figure 5), although a trend to increased collagen production was observed with the highest dose of IL-17E (600 ng/ml) in HD rather than SSc. Overall, our findings indicate that IL-17F and IL-17E, but not IL-17C, promote a pro-inflammatory fibroblast phenotype in normal and SSc individuals with limited impact on collagen production.

## Discussion

Dermal fibrosis as observed in SSc skin and morphea is likely to be the result of complex events in which several cell types and inflammatory mediators participate. It is also possible that different cascades of events may converge into fibrosis, which may then be clinically and histologically monomorphic. Within this framework, the results we report here highlight differences in the frequency of cells positive for various members of the IL-17 cytokine family when fibrotic skin is compared to healthy skin and, maybe more interesting, when comparison is made between clinically affected SSc and morphea skin. We documented, confirming previous findings, that IL-17A+ cells were more abundant in SSc dermis than in HD [15], [23]. Th17 cells contributed only partially to this increased frequency, since most IL-17+ cells also co-stained for tryptase revealing their mast cell origin. Whether SSc mast cells in the skin actively synthetize or simply accumulate IL-17A has not yet been experimentally addressed. Intriguingly, the general frequency of IL-17A+ cells in morphea did not differ from that found in HD skin and was significantly lower than in SSc. This notwithstanding, the cell distribution in the IL-17A+ compartment was similar in both SSc and morphea, with IL-17A+ mast cells outnumbering Th17 cells. What drives IL-17A production in SSc is unclear, although SSc serum levels of cytokines promoting Th17 differentiation, such as IL-6 and IL-23, were reported to be increased [12]. We can speculate that the same events do not take place in morphea. This is consistent with the view that IL-17A in SSc could reflect and participate to systemic autoimmunity including humoral autoimmunity, which is characteristically a minor component in morphea [35]. Our findings highlight the dramatic differences in the expression of IL-17A and IL-17F by cells within the dermis particularly in SSc, but also in morphea and HD. IL-17A and IL-17F are generally considered to be coordinately regulated, however, differences in their production by Th17 cells upon T cell receptor engagement have been documented [33], [34]. Our findings extend these observations. Nonetheless, in our functional assays, IL-17F induced responses in fibroblasts similar to those induced by IL-17A, although higher concentrations were needed, in line with the reported lower potency of IL-17F compared to IL-17A due to lower affinity for their common heterodimeric IL-17RA/IL-17RC receptor [36].

In both morphea and SSc dermis we found, for the first time, an increased frequency of IL-17E+ cells, suggesting that IL-17E may represent a fibrotic-specific cytokine. IL-17E is produced by many cell types of hematopoietic (including ILC) and non-hematopoietic origin. IL-17E is thought to induce and participate to Th2-like responses, which may be mechanically linked to fibrosis development [2], as demonstrated in mice models of pulmonary fibrosis [30]. In our functional assays, IL-17E weakly induced IL-6, MCP-1 and MMP-1, with no effects on type I collagen production, both in HD and SSc fibroblasts. This contrasts with prior evidence indicating that IL-17E induces collagen production in healthy lung fibroblasts [29]. Differences in culture systems or more likely differences in lung and skin-resident fibroblasts may explain this difference. Whether IL-17E could work in conjunction with other profibrotic cytokines including IL-4 and/or IL-13 to enhance fibroblast capacity to produce collagen and other ECM components requires further investigations. Of interest, in our experimental conditions SSc fibroblasts respond to IL-17E by producing more IL-6 than controls.

Unexpectedly, our results document a decreased frequency of IL-17C+ cells in SSc and morphea compared to HD. The concordant lower expression of IL-17C in fibrotic compared to healthy dermis strongly suggests that this is a fibrosis-specific finding. Whether this is cause or consequence, remains to be established. When testing functionally IL-17C on SSc and HD dermal fibroblasts, we did not observe any response. This is consistent with previous evidence indicating lack of response in fibroblast to this cytokine, due to lack of fibroblast expression of the IL-17RE receptor chain needed to respond to IL-17C [27], [37]. IL-17C is known to induce the production of antibacterial peptides and inflammatory mediators by epithelial cells including keratinocytes and to enhance IL-17A production by Th17 cells, with fast induction kinetics [27]. In the perspective of fibrosis development and favoring the hypothesis of a causal relationship between low IL-17C+ cell numbers and fibrosis, it could be hypothesized that IL-17C provides an inhibitory signal to cells that have the capacity to enhance ECM production by fibroblasts. The lack of this inhibitory signal would then indirectly favor fibrosis. This hypothesis needs to be submitted to experimental scrutiny.

From the clinical point of view, it is important to stress that the frequency of IL-17A+ and IL-17F+ cells increases with disease duration, while the frequency of IL-17F+ cells correlates inversely with the extent of skin fibrosis. These observations argue again for a role of IL-17A and IL-17F in restraining rather than enhancing fibrosis and may participate to the well documented, not-treatment related, decrease with time of the extent of skin fibrosis [38].

In conclusion, by applying an immunohistochemistry approach to study the expression of IL-17 isoforms, we found that IL-17E is high and IL-17C low in fibrotic dermis, providing thus evidence of a signature specific to fibrosis. Furthermore, the discordant expression of IL-17A (high in SSc and low in morphea) and IL-17F (low in SSc) underlies disease-specific differences, consistent with the hypothesis that different pathogenic events may converge into fibrosis.
